# Assessing professional behaviour: Overcoming teachers’ reluctance to fail students

**DOI:** 10.1186/1756-0500-7-368

**Published:** 2014-06-17

**Authors:** Marianne Mak–van der Vossen, Saskia Peerdeman, Walther van Mook, Gerda Croiset, Rashmi Kusurkar

**Affiliations:** 1 VUmc School of Medical Sciences, P.O. box 7057, 1007 MB Amsterdam, the Netherlands; 2 LEARN! Research Institute for Learning and Education, VU University Amsterdam, Amsterdam, the Netherlands; 3 VU University Medical Centre Amsterdam, P.O. box 7057, 1007 MB Amsterdam, the Netherlands; 4 Department of Intensive Care Medicine, University Hospital Maastricht, Maastricht, the Netherlands; 5 Department of Medical Education Development and Research, Faculty of Health, Medicine and Life Sciences, Maastricht University, Maastricht, the Netherlands

**Keywords:** Professionalism, Professional behaviour, Faculty development

## Abstract

**Background:**

Developing professional behaviour is an important goal of medical education in which teachers play a significant part. Many teachers can be reluctant to fail students demonstrating unprofessional behaviour. We hypothesize that supporting teachers in teaching and assessing professional behaviour and involving them in remediation will reduce this reluctance.

**Findings:**

In 2010, VUmc School of Medical Sciences Amsterdam introduced an educational theme on professional behaviour for the bachelor's and master's programmes in medicine with a special emphasis on supporting teachers in teaching and assessing professional behaviour and involving them in the remediation process. Information was extracted from the student database on the number of unprofessional behaviour judgments awarded over 2008-2010 (before the intervention), and 2010-2013 (after introducing the intervention), which was compared. To find out if teachers' reluctance to fail had decreased, qualitative feedback from the teachers was gathered and analysed. Since the implementation of the educational theme, the number of unprofessional behaviour judgments has risen. The teachers are positive about the implemented system of teaching and assessing professional behaviour, and feel less reluctant to award an unsatisfactory professional behaviour judgment.

**Conclusions:**

Supporting teachers in teaching and assessing professional behaviour and involving them in students' remediation appears to reduce their reluctance to fail students demonstrating unprofessional behaviour.

## Background

The literature on teaching professional behaviour (PB) suggests that teachers often do not fail students who demonstrate unprofessional behaviour [1,2]. The reasons for this reluctance to fail include lack of time, lack of appropriate documentation, concern for the subjectivity of one’s judgment, fear of being seen as a villain, a lack of commitment to the organizational hierarchy, fear of complaints and lawsuits, and uncertainty about the remediation process and its outcomes [1,3,4]. As teachers often see a PB judgment as a high-stakes decision, they frequently seek confirmation from their colleagues of the student’s past record of unprofessional behaviour [3]. Also, when there is no clear remediation trajectory, failing a student on PB can be perceived as punishment, rather than as an opportunity for remediation [5].

Teachers of VUmc School of Medical Sciences Amsterdam showed a reluctance to fail students, as can be seen by the school’s lower percentage of unprofessional behaviour judgments (1–2%) compared to those reported in the literature (from 5% to 20%) [6]. This paper details measures employed to decrease teachers’ reluctance to fail students on PB, as well as the impact of these measures on the reporting of unprofessional behaviour displayed by students.

## Findings

### Context

VUmc School of Medical Sciences Amsterdam offers a three-year bachelor’s degree (BA) programme and a three-year master’s degree (MA) programme in medicine. In 2010, the school introduced an educational theme on PB for both BA and MA programmes. This particularly emphasised supporting teachers in teaching and assessing PB, as well as involving them in the remediation process of those students assessed as displaying unprofessional behaviour [7]. To ensure continuity in guidance for PB, there is one PB coordinator for both BA and MA programmes.

### Types of unprofessional behaviours and remediation options

Professional behaviour issues identified in this study range from sloppiness, nonchalance and passivity, to insufficient organisational skills or bad communication skills. Although most students involved had good intentions, they often were not sufficiently aware of their behaviour’s impact on teachers, peers and patients. Teachers can remediate these students by immediately giving feedback when the unprofessional behaviour is witnessed, thus making them aware of how their behaviour is perceived by others. Students who lack essential skills, for example in communication or organisation of work, can be offered individual training and opportunities to practice those skills.

### Intervention comprises two concrete measures

#### 1. Supporting teachers

Teachers are supported by providing them with opportunities to consult experts and peers on PB issues. When teachers have doubts about how to guide or assess a student, they can consult the PB coordinator and get advice about what information needs to be collected and what steps need to be taken to arrive at an accurate judgment of a given situation. Additionally, tutors in the BA and MA programmes have the chance to meet regularly (six times a year) in “communities of practice”, in which they can discuss experiences and challenges of teaching and testing PB. Up to 60% of teachers make use of this opportunity [8].

#### 2. Involving teachers in remediation

Both the teacher who gives an unprofessional behaviour judgment and the teacher of that student’s next course are involved in the remediation process.

##### Involving the teacher who awarded an unprofessional behaviour judgment

After an unprofessional behaviour judgment has been made, the PB coordinator meets with the student, together with the teacher who made the assessment. The aim of this discussion is to agree on learning objectives for the student’s next course.

##### Involving the teacher of the student’s next course after a first unprofessional behaviour judgment

The student is strongly advised to discuss his or her learning objectives with the teacher of his or her next course. Teachers of all courses in which PB is evaluated are told to ask for students’ learning objectives. The student can “compensate” for his or her unprofessional behaviour by obtaining a judgment of satisfactory professional behaviour in the subsequent course (in the BA) or by repeating the course or clerkship (in the MA).

### Involving the teacher of the next course after a student’s second unprofessional behaviour judgment

In the case of a second unprofessional behaviour judgment, the PB coordinator and the student have a personal discussion of the student’s learning objectives with the teacher from the next course. This step is called “forward feeding” and is an obligatory part of the process. It aims to make the teacher more able to guide the student toward specific learning goals, as well as to ensure that PB guidance is longitudinal within the programme.

### Analysis

To determine the impact of this intervention, we obtained information from the school’s student database regarding the number of unprofessional behaviour judgments made over 2008–2010 (before the intervention) and 2010–2013 (after the introduction of the intervention). To help understand and interpret the quantitative data, qualitative data were also gathered through feedback from teachers on their willingness to fail students based on unprofessional behaviour. We used unsolicited feedback received from BA tutors for this purpose. Teachers in the MA programme gave feedback through workshops on PB which we organised.

The study has been granted an exemption from requiring ethics approval by the Medical Ethics Review Committee of VU University Medical Center. Oral informed consent was obtained from participants.

## Results

**Figure 1:**
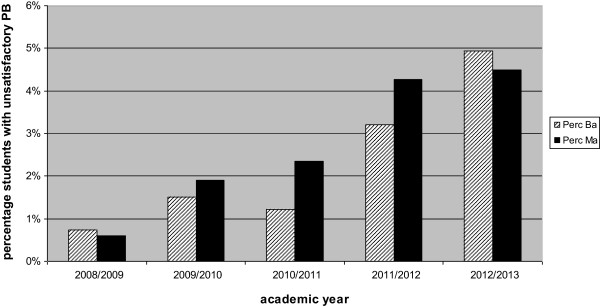
Percentage of students assessed as showing unprofessional behaviour.

The percentage of students who received a summative unprofessional behaviour judgment rose from 0.7% in 2008–2009 to 5.7% in 2012–13 in the BA programme (average number of students/year: 1500) and from 0.6% in 2008–2009 to 4.5% in 2012–2013 in the MA programme (average number of students/year: 1000 – see Figure 1). Qualitative data were collected from 20 BA tutors (from a total number of approximately 100) and 30 MA teachers (from a total number of approximately 150) during workshops.

The teachers expressed positivity about the current system of teaching and assessing PB, and felt supported by the specific attention given to their needs. The teachers also expressed appreciation of the option to contact the PB coordinator before actually awarding a judgment, to discuss or report concerns and receive advice.

In addition, the teachers appreciated being involved in the remediation process and valued their new role in the PB conversation, which is held along with the PB coordinator and the student. When this was not feasible due to practical reasons, they appreciated receiving a report of this conversation. Teachers now realise that PB judgments are a helpful tool to provide students with feedback on their PB, and they are more aware of the importance of their role in developing PB among students. When teachers find that formative assessment feedback does not improve a student’s behaviour, they are now more willing to report unprofessional behaviour in the summative assessment at the end of the course, allowing a student to benefit from the subsequent remediation trajectory.

## Discussion

Comparing the time periods studied, unprofessional behaviour judgments increased between 2008–2010 and 2010–2013, most likely due to the new educational theme concerning PB which was implemented in the school in 2010. Both the BA and MA programmes paid ample attention to supporting teachers in PB assessments and involving them in the subsequent remediation trajectory. The aim was to help teachers overcome their reluctance to award an unsatisfactory judgment even in doubtful cases, and, given the results of this study, this goal appears to have been achieved. However, the percentage of unprofessional behaviour judgments given at the school is still low compared to what is reported in the literature. Thus, this issue deserves further attention (6,10).

Furthermore, to understand the study’s quantitative results better, we gathered feedback from the teachers. This qualitative feedback indicated increasing willingness by the teachers to identify and report unprofessional behaviour. There is no indication that the increased number of unprofessional behaviour judgments is due to teachers’ changing perceptions of PB. Since teachers are now well-informed about a student’s individual remediation trajectory plan, remediation is now seen as a helpful measure for the student, rather than as a punishment. This finding is in line with those of other studies [3,5,9,10].

One limitation of this study is the lack of feedback from students about the newly implemented educational theme, specifically as to whether they perceive forward feeding to be useful in helping them to improve their PB. A second limitation is that qualitative feedback was obtained from a voluntary sample of participating faculty, and hence may not reflect the opinions of all teachers involved in the programme.

## Conclusion

Supporting teachers in teaching and assessing students’ PB and involving them in remediation of unprofessional behaviour helps them to become less reluctant to fail those students who demonstrate unprofessional behaviour.

## Abbreviations

PB: Professional behaviour; BA: Bachelor’s degree programme; MA: Master’s degree programme.

## Competing interests

The authors declare that they have no competing interests.

## Authors’ contributions

All authors have made substantive intellectual contributions to this study. MM carried out the study and the data acquisition, and drafted the manuscript. SP contributed in the conception and design of the study. WvM, GC and RK have been involved in revising the manuscript critically for important intellectual content. All authors read and approved the final manuscript.

## Authors’ information

Marianne Mak-van der Vossen is a general practitioner and medical teacher at VU University Medical Centre, coordinator of the educational theme ”Professional Behaviour”, and a PhD candidate at Research in Education, VUmc School of Medical Sciences, Amsterdam, the Netherlands. Saskia Peerdeman is a neurosurgeon at VU University Medical Centre, and the examiner of “Professional Development”, VUmc School of Medical Sciences, Amsterdam, the Netherlands. Walther van Mook is an internist-intensivist at Maastricht University Medical Centre, and Chair of the Committee on Professional Behaviour, Faculty of Health, Medicine and Life Sciences, Maastricht, the Netherlands. Gerda Croiset is professor of medical education and the director of VUmc School of Medical Sciences, Amsterdam, the Netherlands. Rashmi Kusurkar is the head of Research in Education, VUmc School of Medical Sciences, Amsterdam, the Netherlands.
